# Satisfaction and experience with colorectal cancer screening: a systematic review of validated patient reported outcome measures

**DOI:** 10.1186/s12874-021-01430-7

**Published:** 2021-10-27

**Authors:** A. Selva, C. Selva, Y. Álvarez-Pérez, N. Torà, P. López, R. Terraza-Núñez, V. Rodríguez, I. Solà, on behalf of The CyDESA group, Andrea Burón, Francesc Macià, Salvador Machlab, Carles Pericay, Teresa Puig

**Affiliations:** 1 Clinical Epidemiology and Cancer Screening, Parc Taulí Hospital Universitari, Corporació Sanitària Parc Taulí, Edifici Santa Fè. Parc Taulí 1, Sabadell, 08208 Barcelona, Catalonia Spain; 2 Universitat Autònoma de Barcelona, Bellaterra, Spain; 3 REDISSEC (Health Services Research on Chronic Patients Network), Madrid, Spain; 4 Universitat Oberta de Catalunya (Estudis de Psicologia i Ciències de l’Educació), Barcelona, Catalonia Spain; 5 Fundación Canaria Instituto de Investigación Sanitaria de Canarias (FIISC), Tenerife, Spain; 6 Cancer Screening Programms. Althaia Xarxa Assistencial Universitària de Manresa, Manresa, Catalonia Spain; 7 Direcció General de Planificació en Salut, Departament de Salut, Generalitat de Catalunya, Barcelona, Catalonia Spain; 8 Tecnocampus, Universitat Pompeu Fabra, Mataró, Catalonia Spain; 9 Institute of Biomedical Research, Hospital de la Santa Creu i Sant Pau, Barcelona, Spain; 10 Iberoamerican Cochrane Centre, Hospital de la Santa Creu i Sant Pau, Barcelona, Spain; 11 CIBER Epidemiología y Salud Pública, Instituto de Salud Carlos III, Madrid, Spain; 12 Epidemiology and Evaluation Department, Hospital del Mar, Barcelona, Catalonia Spain; 13 IMIM (Hospital del Mar Medical Research Institute), Barcelona, Catalonia Spain; 14 Gastroenterology Department, Institut d’Investigació i Innovació Parc Taulí I3PT, Parc Taulí Hospital Universitari, Sabadell, Catalonia Spain; 15 Medical Oncology Department, Part Taulí Hospital Universitari, Sabadell, Catalonia Spain

**Keywords:** Patient satisfaction, Patient experience, Colorectal cancer screening, systematic review, Patient reported outcome measures, PROM, Instruments, Questionnaires

## Abstract

**Background:**

Patient satisfaction or experience with colorectal cancer screening can determine adherence to screening programs. An evaluation of validated patient reported outcome measures (PROMs) for measuring experience or satisfaction with colorectal cancer screening does not exist. Our objective was to identify and critically appraise validated questionnaires for measuring patient satisfaction or experience with colorectal cancer screening.

**Methods:**

We conducted a systematic review following the COnsensus-based Standards for the selection of health Measurement INstruments (COSMIN) methodology. We conducted searches on MEDLINE, EMBASE, PsychINFO, CINAHL and BiblioPRO and assessed the methodological quality of studies and measurement properties of questionnaires according to the COSMIN guidelines for systematic reviews of PROMs. PROSPERO registration number: CRD42019118527.

**Results:**

We included 80 studies that used 75 questionnaires, of which only 5 were validated. Four questionnaires measured satisfaction with endoscopy: two in the context of colorectal cancer screening (for colonoscopy and sigmoidoscopy) and two for non-screening endoscopy. One questionnaire measured satisfaction with bowel preparation. The methodological quality of studies was variable. The questionnaires with evidence for sufficient content validity and internal consistency were: the CSSQP questionnaire, which measures safety and satisfaction with screening colonoscopy, and the Post-Procedure questionnaire which measures satisfaction with non-screening endoscopic procedures.

**Conclusions:**

This systematic review shows that a minority of existing PROMs for measuring patient satisfaction with colorectal cancer screening are validated. We identified two questionnaires with high potential for further use (CSSQP and the Post-Procedure questionnaire).

**Supplementary Information:**

The online version contains supplementary material available at 10.1186/s12874-021-01430-7.

## Background

Colorectal cancer is the third most common cancer among men and the second among women and is the second cause of cancer death worldwide [1]. Its 5-year survival rate is 57% for colon cancer and 56% for rectal cancer [2]. Survival is related to tumor stage at diagnosis, so screening strategies have the potential to reduce the burden of the disease through early detection [3, 4].

Colorectal cancer screening aims to detect latent disease in early stages, so it can be treated more effectively than if diagnosed when symptoms appear [5]. Organized screening programs have proven to reduce incidence and mortality from colorectal cancer [5–9]. There are different tests that can be used for colorectal cancer screening: 1. stool tests (guaiac or immunochemical); 2. endoscopic tests (sigmoidoscopy and colonoscopy); 3. image test (CT colonography and capsule endoscopy); and 4. biomarkers in peripheral blood. In Europe, stool tests, particularly fecal immunochemical tests, are the most used in organized screening programs [5, 10, 11]. However, in North America, colonoscopy remains the most commonly used procedure [10].

For organized screening programs to have the expected population impact, it is essential that the participation and adherence rates are high [5]. Patient experience and satisfaction with screening programs are among the factors that determine adherence to them. Studies conducted on colorectal cancer screening showed that satisfaction with past stool test screening is a strong behavioral predictor of adherence to future screening rounds [12–14]. In addition, for breast cancer screening, several studies have shown that perceived satisfaction with screening can lead to good program adherence [15–18]. Furthermore, it should be borne in mind that screening programs are aimed at asymptomatic populations that have not required or requested health care for this condition, and it is the health system itself that invites them to participate. For these reasons, it is necessary to measure and monitor the experience and satisfaction of participants in relation to colorectal cancer screening.

Patient experience and patient satisfaction are patient-reported outcome measures (PROMs) often used interchangeably despite having a small difference in meaning [19, 20]. While patient experience provides a report of the health care from the receiver’s perspective, patient satisfaction involves some sort of rating or evaluation [19]. Although patient satisfaction lacks a formal definition, it can be understood as a subjective evaluation of health care based on the extent to which patients’ expectations are met [20, 21]. Both patient experience and satisfaction have been used to monitor the quality of health care services, benchmark hospital performance and establish hospital rankings, and monitor the effectiveness of interventions [19, 22]. The most used method to obtain these patient-reported measures is self-reported questionnaires. However, these questionnaires need to be valid (they accurately represent the patient experience or satisfaction) and reliable (the measure is consistent) [19, 20].

To our knowledge, an evaluation of validated PROMs to measure patient experience and/or satisfaction with colorectal cancer screening does not exist. Our objective was to identify all the questionnaires used for measuring patient experience or satisfaction with colorectal cancer screening and critically appraise the measurement properties of those validated.

## Methods

We conducted a systematic review following the COnsensus-based Standards for the selection of health Measurement INstruments (COSMIN) methodology for systematic reviews of patient-reported outcome measures (PROMs) [23–25]. We registered the review protocol in PROSPERO (http://www.crd.york.ac.uk/PROSPERO) [registration number CRD42019118527] and report its findings according to the PRISMA statement [26]. This systematic review is part of a broader project, the CyDESA study that aims to evaluate satisfaction and patient participation in decision making in colorectal cancer screening.

### Search strategy

We conducted an exhaustive search in MEDLINE (PubMed), EMBASE (Ovid), PsycINFO (Ovid) and CINAHL (EBSCOHost) without language or date restrictions. We kept the search updated while we conducted the review and performed the last search in October 2020. The detailed search strategies and dates are available in Annex A. We also searched in BiblioPRO and checked the references listed in included studies. We designed a search strategy combining controlled vocabulary from each database and text words related to the topics review (e.g., satisfaction and colorectal screening). Although a proposal to find studies on PROMs measurement properties exists [27], we defined and used a more specific list of terms to filter the search results.

### Eligibility criteria

We included validation studies which reported the development and/or the evaluation of one or more measurement properties of questionnaires measuring patient experience or satisfaction with colorectal cancer screening, irrespective of the screening test used. To avoid being too restrictive, we also included studies on the development or validation of questionnaires that measure patient experience or satisfaction with colonoscopy (irrespective of it was performed in the context of a screening program) and with the notification process of a screening result. We also considered studies (irrespective of their design) that assessed patient experience or satisfaction with colorectal screening as an outcome. From these studies, we tried to obtain information on the questionnaire used to measure the outcome and tried to locate the validation study to consider its inclusion.

We limited the inclusion to studies published in English, Spanish, French and Italian. We excluded studies that assessed satisfaction with the decision to uptake screening and studies that used alternative methods to questionnaires to measure experience or satisfaction, such as interviews or diaries.

Two authors independently assessed the results of the search for eligibility, and then made a final decision based on the full text of the references deemed eligible. Disagreements were resolved with the help of a third reviewer.

### Data extraction

We developed and pilot-tested a case report form (CRF) using Google Forms. The CRF is available from the authors on request. Two authors independently extracted data from included studies and disagreements were resolved with the help of a third reviewer. When full questionnaires were not reported in the paper, we tried to contact the corresponding authors in order to obtain them.

We extracted the following data from eligible documents following the recommendations from the COSMIN user manual [23, 28]: 1. General characteristics of the study (country, year of publication, study design, objective, main outcomes); 2. Characteristics from the questionnaire targeted population or those that participated in the validation; 3. Main characteristics of the questionnaire (name, original language and available translations, administration characteristics, domains measured, number of items, evidence for validity); 4. Information on questionnaires psychometric properties; 5. Information on interpretability of questionnaires (the degree to which a quantitative score or a change in score of a questionnaire can have a qualitative meaning) and feasibility (the ease of application of the questionnaire in a setting). Interpretability and feasibility are not considered measurement properties, but are important aspects for selecting a questionnaire to use in practice [23].

### Assessment of methodological quality of included studies

We assessed the methodological quality of each measurement property study using the COSMIN Risk of Bias checklist [24]. According to this checklist, methodological quality of studies was rated as either “very good”, “adequate”, “doubtful” or “inadequate” for each measurement property assessed. We used the COSMIN taxonomy to determine which measurement property were assessed in each study.

### Assessment of measurement properties results

The result of each measurement property study was rated against the updated criteria for good measurement properties based on Terwee et al. [29] and Prinsen et al. [30] (Annex B). Each result was rated as either sufficient (+), insufficient (−), or indeterminate (?).

Following the COSMIN manual, we graded the quality of the evidence for the rating of each measurement property of each questionnaire using the GRADE approach [31], which specifies four levels of quality of evidence (high, moderate, low or very low) depending on the presence of four factors (risk of bias, indirectness, inconsistency and imprecision). If the overall rating for a measurement property is indeterminate (?), the quality of the PROM cannot be judged and there will be no grading of the quality of the evidence [23].

The process of assessing methodological quality of studies, rating measurement properties and grading the evidence was done by two authors independently and differences were resolved by consensus. Attempts were made to contact the authors of included PROMs for information on all measurement properties of questionnaires.

According to ratings on methodological quality and the results of measurement properties, included PROMs were classified providing a recommendation on the most suitable questionnaire to be used [23] (Table 1).

**Table 1:** Categories for classification of PROMs

Category	Characteristics	Implications
A	Evidence for sufficient content validity (any level) and at least low evidence for sufficient internal consistency	Have potential to be recommended as the most suitable questionnaire for the construct and population of interest
B	Questionnaire not categorized in A or C	May have the potential to be recommended, but further validation studies are needed
C	High quality evidence for an insufficient measurement property	Should not be recommended

### Data analysis and synthesis

We used descriptive statistics to synthesize findings, calculating absolute frequencies and proportions as appropriate. We planned to quantitatively pool the results reported by different studies on measurement properties of each questionnaire. However, we were not able to do so as we only found one study for each questionnaire. A statistical analysis was performed using SPSS, version 25.0 (SPSS Inc., Chicago, IL, USA). We report the findings of the review as a narrative synthesis of the characteristics and measurement properties from each included questionnaire.

### Ethical approval

No ethical approval was required as this study is a systematic review.

## Results

### Study selection

**Fig. 1:**
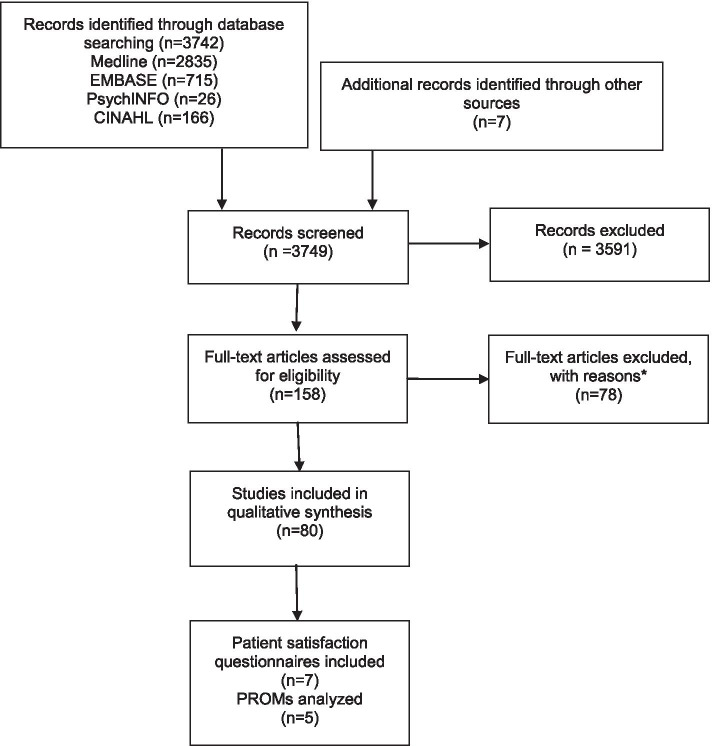
PRISMA 2009 Flow Diagram. *Reasons for exclusion: Not measures satisfaction with colorectal cancer screening (*n* = 46); Not uses a questionnaire (*n* = 13); Narrative review (*n* = 6); Measures satisfaction with the decision to participate in a study (*n* = 3); Not about colorectal cancer screening (*n* = 2); Study protocol (n = 1); Language (n = 1), Duplicate (n = 4), Measures satisfaction in relation to a small part of the process (use of a reminder letter, sedation protocol used, n = 2). PROMs: patient reported outcome measures

We describe the eligibility process in a PRISMA flowchart [26] (Fig. 1). We screened the titles and abstracts of 3749 references obtained from the searches, selected 158 records for full-text assessment and finally included 80 studies. Reasons for exclusions are detailed in Fig. 1.

### Characteristics of included studies and questionnaires

We identified 80 studies published from 1992 to 2020 that used questionnaires to measure patient experience or satisfaction with colorectal cancer screening or with the conduction of non-screening colonoscopy, sigmoidoscopy or with bowel preparation. Most of them were published in North America (38, 47.5%) and Europe (26, 32.5%) from 2010 on (Table 2). Most studies were experimental or observational studies in which patient experience or satisfaction were measured as outcomes (75, 93.7%) and only five studies (6.2%) described the development of a questionnaire or its validation. These 80 studies used 75 different questionnaires, being most of them self-administered (70, 93.3%) and written in English (61, 81.3%). Most were created de novo (51, 68.0%) or developed from existing questionnaires (12, 16.0%), with very few studies using an existing questionnaire (8, 10.7%).

**Table 2:** Characteristics of included studies and questionnaires

Included studies (*n* = 80)	N	%
Continent		
North America	38	47.5
Europe	26	32.5
Asia	8	10.0
Oceania	6	7.5
Other	2	2.5
Year of publication		
< 2005	24	30.0
2005–2010	14	17.5
> 2010	42	52.5
Study design		
Experimental or quasi-experimental	28	35.0
Other study designs, patient experience/satisfaction is an outcome	47	58.7
Study on the development or validation of a questionnaire	5	6.2
Administration of questionnaires^a^	75	
Self-reported	70	93.3
By telephone	3	4.0
Not reported	2	2.7
Availability of the questionnaire^a^		
Yes	32	42.7
No	43	57.3
Original language of the questionnaire^a^		
English	61	81.3
Spanish	4	5.3
Chinese	2	2.7
Korean	2	2.7
Other	6	8.0
Origin of the questionnaire used^a^		
Development of a new questionnaire for the study	51	68.0
Derived from an existing questionnaire	12	16.0
Use of an existing questionnaire	8	10.7
Not reported	4	5.3
Aspects/interventions assessed^a^		
Bowel preparation	11	14.7
Bowel relaxant	1	1.3
Colonoscopy	32	42.7
Diet	1	1.3
Stool test	13	17.3
Pre-colonoscopy consultation	2	2.7
Sigmoidoscopy	12	16.0
CT-colonography	2	2.7
Whole screening program	1	1.3

^a^*n* = 75 questionnaires in 80 studies

### Characteristic of validated questionnaires

From the 75 questionnaires identified, only seven (9.3%) were reported as validated tools [32–38]. There were two studies that reported using validated questionnaires, but we were unable to locate the development or validation studies or to obtain a copy of them (although an effort was made to contact authors) [37, 38]. For that reason, we could only analyze five validated questionnaires (6.6%) [32–36].

All five validated questionnaires measured patient satisfaction. Four questionnaires assessed satisfaction with endoscopic procedures (Table 3). Two were developed in the context of colorectal cancer screening (the Colonoscopy Satisfaction and Safety Questionnaire (CSSQP) for colonoscopy conducted after a positive stool test [32] and the Screening Flexible Sigmoidoscopy Assessment Questionnaire for screening sigmoidoscopy [36]). The other two measured satisfaction in the context of elective upper or lower endoscopy (the Spanish modified Group Health Association of America- 9 Questionnaire (SmGHAA-9 m) [34] and the Post Procedure Questionnaire [35]). The remaining questionnaire assessed satisfaction with bowel preparation for colonoscopy [33].

**Table 3:** Characteristics of validated questionnaires

Questionnaire (Reference)	Country	Language/ translation	Mode of administration	Intervention assessed	Construct (according to authors)	Dimensions/Domains	Number of items
CSSQP Brotons 2019^32^	Spain	Spanish English translation	Self-reported	Colonoscopy after a positive fecal occult blood test in colorectal cancer screening	1. Satisfaction 2.Safety	1. Satisfaction scale: -Information -Care -Service and facilities 2. Safety scale -Information gaps -Safety incidents	-Satisfaction scale: 15 -Safety scale: 3
Patient Satisfaction Scale with Bowel Preparation Hatoum 2016^33^	USA	English No translation	Self-reported	Bowel preparation	1.Satisfaction with bowel preparation 2. Acceptance or refusal of future use of the preparation	1. Current satisfaction: -Difficulty using bowel-cleansing preparations -Ability to consume preparations -Acceptability of taste -Overall experience 2. Acceptance or refusal of future use of the same bowel preparation	6
Post procedure endoscopy questionnaire Peña 2005^35^	USA	English Translation not reported	Self-reported	Gastrointestinal endoscopy (upper and lower)	Satisfaction	1. Anxiety 2. Pain or discomfort 3. Distress or suffering 4. Physical needs met 5. Emotional needs met 6. Overall satisfaction 7. Willingness to repeat if necessary	7
SmGHAA-9 m Sánchez del Río 2005^34^	Spain	Spanish English translation	By telephone by an interviewer	Gastrointestinal endoscopy (upper and lower)	Satisfaction	1.Waiting times 2. Personal manners 3. Information received 4. Discomfort 5. Overall rating 6. Willingness to repeat if necessary	7
Screening Flexible Sigmoidoscopy Assessment Questionnaire. Schoen 2000^36^	USA	English Translation not reported	Self-reported	Screening sigmoidoscopy	Satisfaction	1. Convenience and accessibility 2. Staff interpersonal skills 3. Physical surroundings 4. Technical competence 5. Pain and discomfort 6. Expectations and beliefs 7. General satisfaction	18

Three questionnaires used the English language and were developed and validated in the USA [33, 35, 36], while two were developed and validated in Spain and used the Spanish language although an English translation is available [32, 34]. All questionnaires were self-administrated with the exception of the SmGHAA-9 m [34] which was administered by telephone.

The questionnaires were validated in samples of women and men between 50 and 69 years old, with the exception of the Patient Satisfaction Scale with Bowel Preparation and the Post-Procedure endoscopic questionnaire [33, 35], which were evaluated in adults up to 80 years old. Table 4 describes the characteristics of included populations.

**Table 4:** Characteristics of the included study populations

Questionnaire (Reference)	Population			Disease characteristics	Questionnaire administration		
	N	Age Mean (SD) Range (%)	Gender	Disease	Setting	Moment of administration	Response rate
CSSQP^32^	505	60.7 (5.2)	38.6% female	Women and men who had undergone a colonoscopy after a positive fecal occult blood test within the colorectal cancer screening program	2 hospitals	The day after the colonoscopy and return to a mailbox	74.9% (378/505)
Patient Satisfaction Scale with Bowel Preparation^33^	1.211	56	61% female	Women and men (18–80 years) scheduled for an elective outpatient colonoscopy	University hospitals, academic medical centers, private clinics	Prior to the colonoscopy	98.7% (1195/1211)
Post- procedure questionnaire^35^	148	21–40 (18%) 41–60 (64%) 61–80 (17%) > 80 (1%)	63% female	Women and men (≥18 years) who underwent routine procedures (gastric endoscopy, colonoscopy, sigmoidoscopy)	1 hospital	After the procedure	89,7% (148/165)
SmGHAA-9 m^34^	485	51 (16)	57% female	Women and men scheduled for an endoscopic procedure (colonoscopy or gastroscopy)	2 hospitals: one private (the source of most patients)	By telephone 3 weeks after the procedure	93,8% (455/485)
Screening Flexible Sigmoidoscopy Assessment Questionnaire^36^	1.221	61.8 (6.1)	45.5% female	Women and men who underwent screening flexible sigmoidoscopy	2 hospitals. 97% of participants were participants in an RCT	After the sigmoidoscopy, prior to discharge or returned by mail	100%

### Methodological quality of studies

Methodological quality of studies on each measurement property was evaluated according to the COSMIN Risk of Bias checklist [24] (Table 5). Ratings are provided for only those measurement properties assessed in each study.

**Table 5:** Quality of studies on measurement properties

Measurement property			CSSQP^32^	Patient Satisfaction Survey^33^	Post procedure questionnaire^35^	SmGHAA-9 m^34^	Screening Flexible Sigmoidoscopy Assessment Questionnaire^36^
Content validity	Asking patients	Relevance	D	D	D	D	–
		Comprehensiveness	D	D	D	D	–
		Comprehensibility	D	D	D	D	D
	Asking experts	Relevance	–	D	D	–	D
		Comprehensiveness	–	D	D	–	D
Internal structure	Structural validity		A	–	A	–	A
	Internal consistency		V	D	V	D	V
	Cross-cultural validity		–	–	–	–	–
Other measurement properties	Reliability		–	–	–	I	A
	Measurement error		–	–	–	–	A
	Criterion validity		–	–	–	–	–
	Construct validity	Convergent validity	–	D	–	–	V
		Known groups validity	V	–	–	–	–
	Responsiveness	Comparison with gold standard	–	–	–	–	–
		Comparison with other instruments	–	–	–	–	V
		Comparison between subgroups	–	–	–	–	–
		Comparison before and after intervention	–	–	–	–	–

*V* very good; *A* adequate; *D* doubtful; I: inadequate

Cells not colored correspond to measurement properties not assessed in included studies

According to the COSMIN guidelines, content validity is the most important measurement property [23] and it arises from the assessment of the relevance, comprehensiveness and comprehensibility of the PROM. Evidence on these parameters comes from development and validation studies. A detailed evaluation of the quality of questionnaires’ development studies is available in Annex C. Methodological quality of studies for content validity was rated as doubtful for all questionnaires because it was not clear if patients and experts were asked about relevance, comprehensiveness, and comprehensibility in the validation studies.

Structural validity and internal consistency address the internal structure of a questionnaire, and are the next most important measurement properties [23]. Three studies were of adequate quality for structural validity and of very good quality for internal consistency [32, 35, 36]. The remaining two were of doubtful quality for internal consistency [33, 34]. Methodological quality of studies on remaining measurement properties is summarized in Table 5.

In Annex D we provide an example on how the methodological quality evaluation and the rating of measurement properties were conducted for one questionnaire.

### Measurement properties of PROMs

#### PROMs measuring satisfaction with screening endoscopy

There were two questionnaires assessing satisfaction with screening endoscopic procedures. We gave a COSMIN category A to the CSSQP questionnaire [32] which measures safety and satisfaction with a colonoscopy performed after a positive stool test for colorectal cancer screening. It has sufficient content validity (moderate quality of evidence), sufficient internal consistency with a Cronbach’s alfa ≥0.7 (high quality of evidence), indeterminate structural validity because a confirmatory factor analysis was not conducted, and indeterminate construct validity (Table 6).

**Table 6:** Summary of measurement properties of questionnaires

Questionnaire	Context of use	Measurement property	Methodolo-gical quality^a^	Rating^b^	Quality of Evidence	Recommen-dation^c^
CSSQP^32^	Screening colonoscopy	Content validity	Doubtful	(+) Based on review ratings. Development and validation study not provide enough information to judge relevance, comprehensiveness or comprehensibility	Moderate Serious RoB (content validity and development study of doubtful quality)	A
		Structural validity	Adequate	(?) A confirmatory factor analysis was not conducted	*	
		Internal consistency	Very good	(+) Cronbach’s alpha 0.86 (≥0.7) Spearman-Brown coefficient 0.85	High No RoB	
		Construct validity	Very good	(?) No hypothesis defined	High No RoB	
Patient Satisfaction Scale with Bowel Preparation^33^	Bowel preparation	Content validity	Doubtful	(−) Relevance doubtful, comprehensiveness (−) and comprehensibility (−) as patients and professionals were not asked	Low Very serious RoB (no content validity study, development study of doubtful quality)	B
		Internal consistency	Doubtful	(+) Cronbach’s alpha 0.79 (≥0.70)	Low Very serious RoB (one study of doubtful quality)	
		Construct validity	Doubtful	(?) Results in accordance with hypothesis, associated with narra-tives, but no correlations calculated	*	
Post-procedure questionnaire^35^	Upper and lower endoscopy	Content validity	Doubtful	(+) Relevance, comprehensiveness and comprehensibility were (+)	Low Serious RoB (content validity and development study of doubtful quality) and indirectness	A
		Structural validity	Adequate	(?) No results of exploratory factor analysis	*	
		Internal consistency	Very good	(+) Cronbach’s alpha ≥0.7 for 4 of 8 items analyzed	Low Serious RoB (one study of adequate quality) and indirectness	
SmGHAA-9 m^34^	Upper and lower endoscopy	Content validity	Inadequate	(−) Relevance, comprehensiveness, and comprehensibility rated (−)	Very low Serious RoB (no content validity study and development study of inadequate quality). Indirectness	B
		Internal consistency	Doubtful	(+) Cronbach’s alpha ≥0.7	Very low Very serious RoB (one study of doubtful quality) and indirectness	
		Reliability	Inadequate	(+) Weighted kappa of 0.78	Very low Extremely serious RoB (one study of inadequate quality) and indirectness	
Screening Flexible Sigmoidoscopy Assessment Questionnaire^36^	Screening sigmoidoscopy	Content validity	Doubtful	(+/−) Relevance (+) by reviewers, comprehensiveness (−) and comprehensibility (+/−)	Low Serious RoB (content validity and development studies of doubtful quality) and indirectness	B
		Structural validity	Adequate	(?) Comparative fit index, Tucker-Lewis index, Root Mean Square Error of approximation or Standardized root mean residuals not reported	*	
		Internal consistency	Very good	(+) Cronbach’s alpha 0.87 for overall satisfaction and 0.84 for pain and discomfort scale	Moderate No serious RoB but indirectness	
		Reliability	Adequate	(+) Pearson correlation coefficient 0.82 (≥0.7)	Low Serious RoB (only one study of adequate quality) and indirectness	
		Measurement error	Adequate	(?) Minimal important change not defined	*	
		Construct validity	Very good	(?) Results in accordance with hypothesis and associated with narratives, but no correlations calculated	*	
		Responsiveness	Very good	(+) responses in accordance to narratives	Moderate No RoB but indirectness	

*RoB* Risk of bias

^a^ Assessed according to the COSMIN Risk of Bias checklist [23–25]: each measurement property was assigned a rating of “very good”, “adequate”, “doubtful”, “inadequate” or “not applicable”

^b^ Psychometric properties were rated according to the updated criteria for good measurement properties based on Terwee et al. [14] and Prinsen et al. [30]. (Annex B) Ratings can be:“+” = sufficient,” – “= insufficient, “?” = indeterminate, or “+/” inconsistent

^c^ Recommendations: A: Have the potential to be recommended as the most suitable questionnaire fo the construct and population of interest;B: May have the potential to be recommended, but further validation studies are needed; C:Should not be recommended

*In case the overall rating is indeterminate (?), it is not possible to judge the quality of the instrument, and there is no grading of the quality of the evidence [23]

We gave a COSMIN category B to the Screening Flexible Sigmoidoscopy Assessment Questionnaire [36], which measures satisfaction with screening sigmoidoscopy. It has inconsistent content validity (low quality of evidence), indeterminate structural validity because results of the confirmatory factor analysis were not reported and construct validity. It has sufficient internal consistency with a Cronbach’s alfa ≥0.7 (moderate quality of evidence) and reliability with a Pearson correlation coefficient ≥ 0.7 (low quality of evidence), but an indeterminate measurement error because minimal important change was not defined. Responsiveness was sufficient (moderate quality of evidence).

#### PROMs measuring satisfaction with non-screening endoscopy

There were two questionnaires assessing non-screening endoscopic procedures, both upper and lower. The post-procedure questionnaire [35] has sufficient content validity (low quality of evidence), and internal consistency with a Cronbach’s alfa ≥0.7 (low quality of evidence) but indeterminate structural validity as results of the exploratory factor analysis were not reported. It was classified as A.

The SmGHAA-9 m [34] has insufficient content validity (very low quality of evidence), sufficient internal consistency with a Cronbach’s alfa ≥0.7 (very low quality of evidence) and sufficient reliability with a weighted kappa of 0.78 (very low quality of evidence). It was classified as B.

#### PROMs measuring satisfaction with bowel preparation

The Patient Satisfaction Scale with Bowel Preparation [33] was the only questionnaire identified that assessed satisfaction with bowel preparation. It has insufficient content validity (low quality of evidence), indeterminate construct validity and sufficient internal consistency with a Cronbach’s alfa ≥0.7 (low quality of evidence). This questionnaire was classified as B.

### Interpretability and feasibility

Detailed information on interpretability and feasibility of questionnaires is summarized in Annex E and F**.** Overall, studies provided scarce information about interpretability: most showed a low percentage of missing total scores (from 1.4 to 6.2%) [32–34, 36] but only two provided information on floor and ceiling effects [32, 33] and none on the minimal important change or minimal important difference. Regarding feasibility aspects, none of the studies provided information on the completion time, the cost of the questionnaire or the existence of copyright. The CSSQP [32] and the SmGHAA-9 m [34] are available in Spanish and in English. However, none of these questionnaires were culturally adapted nor validated in a setting different to that in which they were created (cross-cultural validation).

## Discussion

### Main findings

This systematic review identified many studies that measured patient satisfaction or experience with colorectal cancer screening or procedures and tests included in these preventive programs. These findings mean that patient reported measurements are increasingly being considered in this setting and other research fields as well [20]. However, the majority of these studies used non-validated questionnaires, which is consistent with findings of another systematic review of PROMs on patient satisfaction in breast cancer screening [39]. This shows clear room for improvement since the use of non-validated PROMs may result in a limited trustworthiness in measurements obtained through their use.

Only five questionnaires have been validated for measuring patient satisfaction in relation to screening colonoscopy and sigmoidoscopy [32, 36], non-screening endoscopy (both upper and lower) [34, 35] and with bowel preparation [33]. Although two additional studies reported the use of validated questionnaires [37, 38], we were unable to obtain the required information to appraise them. We did not identify any validated questionnaire for assessing satisfaction or experience with the use of stool tests for colorectal cancer screening, which is the most used screening test in European screening programs [5, 10], or with other aspects of the screening process such as the communication of screening results.

The decision to use one PROM over another will depend on different factors, but it is important to ascertain both the methodological quality of studies in which the PROMs were validated and the questionnaire measurement properties themselves. We used the COSMIN methodology to classify each PROM into three possible categories that have different implications regarding the potential to recommend one PROM over another. From the five validated questionnaires included, only two (CSSQP [32] and Post-procedure questionnaire [35]) showed sufficient content validity and internal consistency to be recommended for their use in practice.

There are additional important factors to consider when choosing a PROM. For example, the population for which the PROM is intended, the availability of cross- cultural validation of the questionnaire and aspects related to its feasibility. The CSSQP [32] and the post-procedure questionnaire [35] measure satisfaction with different procedures and are targeted at different populations. The CSSQP [32] assesses the safety and satisfaction with colonoscopy conducted after having a positive stool test in the context of colorectal cancer screening. On the other hand, the post-procedure questionnaire [35] measures satisfaction with both upper and lower non-screening endoscopy. These differences in population are important as screening program attendees have peculiarities with respect to those who attend colonoscopy for other factors (e.g., evaluation of symptoms, surveillance of polyps, etc.). They are healthy people with no symptoms that have not sought health care and may experience high levels of anxiety [40], so their expectations (and therefore their satisfaction) may differ from the rest of patients [32]. Another important aspect is that the CSSQP [32] was developed and validated in the Spanish population. Although this questionnaire was translated to English following a translation back-translation process, it still has not been culturally adapted nor validated in other populations. In the same way, the post-procedure questionnaire [35] was developed and validated in a population from the USA and is only available in English. If questionnaires are used in countries other than those in which they have been developed and validated, it is necessary to translate them (with a translation-back translation process), conduct a culturally adaptation and finally study their cross-cultural validity before their use [28, 41–44]. Cross-cultural validity is evaluated assessing whether the scale is measurement invariant or whether differential item functioning occurs between at least two culturally different groups of people [23]. None of the included questionnaires were culturally adapted and neither conducted this sort of validation.

The remaining three questionnaires [33, 34, 36] did not report data in enough detail to ascertain their validity. It does not mean that these questionnaires cannot be recommended, but further validation studies will be needed [23].

### Our results in the context of previous research

To our knowledge, this is the first systematic review to identify and assess PROMs for measuring patient satisfaction and experience with colorectal cancer screening. However, there are hundreds of systematic reviews using the COSMIN methodology. Some have focused on screening [45–48], and one specifically assessed PROMs for measuring patient satisfaction or experience in relation to breast cancer screening [39]. These systematic reviews also found variability in the methodological quality of included studies [19, 39, 45, 46, 48]. In our work, methodological quality of studies ranged from inadequate to very good, depending on the measurement property assessed.

We were able to assess limited information about some (but not many) psychometric properties of questionnaires, which is consistent with results of similar literature reviews [19, 39]. However, unlike other settings in which there are some studies assessing each PROM, we only found one study for each questionnaire reporting data related to its development and validation. We did not find further validation studies, which limits the available evidence on the questionnaires’ validity, as each new study provides further confirmation of the ability of a questionnaire to measure the construct of interest [19].

Another important aspect is that patient satisfaction and patient experience, despite being related, are not exactly the same [19, 20, 49]. Some authors advocate that measuring patient experience is preferred because it is a more descriptive and objective measure (rather than evaluative) and is less affected by gratitude bias and other factors [19, 20, 49]. As these concepts have been used interchangeably many times in the literature [50], we included both so that important information was not left out. However, all validated questionnaires included measured patient satisfaction.

### Limitations and strengths

We conducted an exhaustive search including sources that index questionnaires and measurement instruments for the identification of all available PROMs, but the possibility of selection bias still exists as we limited the inclusion to studies published in English, Spanish, French and Italian, and we did not look at grey literature to check the use of questionnaires in technical reports assessing the results from local or national screening programs. Despite this drawback, we could expect that, at least for validated PROMs, the researchers made the effort to report the process in a journal article. It is also possible that we did not evaluate all measurement properties of questionnaires because this information was not reported in published papers. In order to minimize this bias, we attempted to contact questionnaire developers for further information and complete scoring. However, we did not always receive an answer. It is possible that included questionnaires have been harshly criticized in their methodological quality as the COSMIN methodology considers applying the lowest rating of any standard in the box to the overall rating of each study (“the lowest score counts” principle) [24].

This study has several strengths. To our knowledge, this is the first paper that reviews and appraises available PROMs to measure patient experience or satisfaction with colorectal cancer screening. We conducted systematic searches in four different databases and the selection and data abstraction processes were conducted in duplicate in order to minimize selection bias and errors. We used an internationally agreed and explicit methodology (COSMIN [23]) for assessing the methodological quality of studies and questionnaires’ measurement properties. We must highlight, though, that the use of this guidance is limited to trained and skilled researchers and that its use is burdensome at some stages. There is clear room for improvement for reporting in this field. Recently, guidelines for reporting primary studies on measurement properties have been published [51] and their use should improve transparency and facilitate the appraisal of these studies. On the other hand, reporting guidelines for reviews of PROMs would be of great interest to ease the preparation of manuscripts in this field and improve the quality of such evidence syntheses.

### Implications for practice and research

This systematic review will help clinicians, managers, policy makers and researchers to select the most suitable PROM taking into consideration their context of use. This will, in turn, facilitate the systematic use of these validated questionnaires to identify areas for enhancement from the patients’ perspective and drive improvements in the quality of colorectal cancer screening programs.

Some validated questionnaires with good measurement properties for measuring patient satisfaction with screening and non-screening colonoscopy are already available, so it is not necessary to develop new questionnaires on this aspect [32, 35]. Efforts should be made in conducting further validations of existing questionnaires (assessing all psychometric properties), in translating them into different languages and validating them in different populations, so they could be used in different settings. However, for measuring satisfaction with bowel preparation or screening sigmoidoscopy, we could identify only one validated questionnaire for each procedure, with not sufficient psychometric properties for it to be recommended for use. In these cases, it would be necessary to conduct further validation studies or even develop new PROMs. Likewise, we did not identify any validated PROM for assessing patient experience or satisfaction with the use of stool tests for colorectal cancer screening or with the communication of screening results, so it would be necessary to develop and validate PROMS to measure these aspects. Any additional effort to develop new PROMs in this field should ensure the involvement of the public in their development and validation [52].

## Conclusion

Only a minority of PROMs used for measuring patient satisfaction with colorectal cancer screening or procedures related to it are validated. Questionnaires vary in their measurement properties and methodological quality and are designed for different settings and populations. The CSSQP questionnaire may be the most suitable questionnaire for measuring satisfaction with screening colonoscopy in Spanish population [32]. For the North American population, the Post-procedure questionnaire may be more suitable, despite being designed to measure satisfaction with non-screening endoscopy [35]. Satisfaction with other aspects of colorectal screening process (use of stool tests, bowel preparation, screening flexible sigmoidoscopy, communication of screening results) need new validation studies of available questionnaires or even the development of new PROMs.

## Supplementary Information

**Additional file 1.**

## Data Availability

The datasets used and/or analyzed during the current study are available from the corresponding author on reasonable request.
