# Supplementary material for: Satisfaction and experience with colorectal cancer screening: a systematic review of validated patient reported outcome measures
Source: BMC Med Res Methodol. 2021 Oct 27;21:230. doi: 10.1186/s12874-021-01430-7 (PMC8549248; doi:10.1186/s12874-021-01430-7)
Supplement: Supplementary file 1 — Additional file 1. [file 12874_2021_1430_MOESM1_ESM.docx]

## Annex A. SEARCH STRATEGIES.

**EMBASE Ovid Embase <1974 to 2019 January 07>08/01/2019**

**Updated in 19/10/2020**

1     exp patient satisfaction/ (124904)

2     satisfaction.ti,ab. (163806)

3     satisfied.ti,ab. (60011)

4     patient experience.ti. (1792)

5     patients experience.ti. (1291)

6     participant experience.ti. (18)

7     participants experience.ti. (18)

8     preference*.ti. (29094)

9     discomfort.ti,ab. (60416)

10     burdensome.ti,ab. (4396)

11     acceptab*.ti. (10290)

12     willing*.ti. (4415)

13     1 or 2 or 3 or 4 or 5 or 6 or 7 or 8 or 9 or 10 or 11 or 12 (356424)

14     exp screening/ (639698)

15     exp early diagnosis/ (97373)

16     screening.ti,ab. (644932)

17     early detection.ti. (12278)

18     14 or 15 or 16 or 17 (1025854)

19     exp colorectal cancer/ (157306)

20     exp colonoscopy/ (71036)

21     colorectal.ti. (115412)

22     f?ecal occult blood test.ti,ab. (2843)

23     f?ecal immunochemical test.ti,ab. (881)

24     stool.ti,ab. (48897)

25     FOBT.ti,ab. (2294)

26     gFOBT.ti,ab. (241)

27     colonoscop*.ti,ab. (51604)

28     colonography.ti,ab. (2696)

29     sigmoidoscopy.ti,ab. (5718)

30     19 or 20 or 21 or 22 or 23 or 24 or 25 or 26 or 27 or 28 or 29 (293343)

31     13 and 18 and 30 (1218)

32     exp psychometry/ (83282)

33     exp patient health questionnaire/ (6676)

34     exp validation study/ (74384)

35     questionnaire*.ti,ab. (650013)

36     psychometr*.ti,ab. (49871)

37     measure.ti,ab. (645843)

38     measures.ti,ab. (843731)

39     instrument*.ti,ab. (326232)

40     tool.ti,ab. (573473)

41     tools.ti,ab. (293006)

42     item.ti,ab. (109821)

43     items.ti,ab. (142847)

44     scale.ti,ab. (825557)

45     scales.ti,ab. (148423)

46     subscale*.ti,ab. (54328)

47     validation.ti,ab. (248124)

48     validity.ti,ab. (186391)

49     reliability.ti,ab. (177108)

50     internal consistency.ti,ab. (30211)

51     convergent.ti,ab. (29763)

52     discrimina*.ti,ab. (284953)

53     construct.ti,ab. (143846)

54     32 or 33 or 34 or 35 or 36 or 37 or 38 or 39 or 40 or 41 or 42 or 43 or 44 or 45 or 46 or 47 or 48 or 49 or 50 or 51 or 52 or 53 (4138991)

55     31 and 54 (608) [hits related to instruments]

56     31 not 55 (610) [hits related to satisfaction as a measurable domain]

**MEDLINE PubMed 01/01/2019**

**Updated in 19/06/2020**

#1        “Patient Satisfaction”[Mesh]  81862

#2        “Patient Acceptance of Health Care”[Majr]  71390

#3        satisfaction[tiab]        118753

#4        satisfied[tiab] 43196

#5        patient experience[tiab]        4535

#6        patients experience[tiab]       7878

#7        participant experience[tiab]  115

#8        participants experience[tiab] 161

#9        experience*[ti]            231320

#10      preference*[ti]            26170

#11      discomfort[tiab]         40894

#12      burdensome[tiab]      2851

#13      acceptab*[tiab]          158798

#14      willing*[tiab]   35942

#15      #1 OR #2 OR #3 OR #4 OR #5 OR #6 OR #7 OR #8 OR #9 OR #10 OR #11 OR #12 OR #13 OR #14            718549

#16      “Mass Screening”[Mesh]        118859

#17      “Early Detection of Cancer”[Mesh]    19901

#18      screening[tiab]           472538

#19      early detection[ti]       9400

#20      #16 OR #17 OR #18 OR #19  527457

#21      “Colorectal Neoplasms”[Mesh]         185893

#22      “Colonoscopy”[Mesh]            27891

#23      colorectal[ti]   79335

#24      fecal immunochemical test[tiab]       393

#25      faecal immunochemical test[tiab]     162

#26      fecal occult blood test[tiab]  1382

#27      stool[tiab]       33165

#28      FOBT[tiab]      1254

#29      gFOBT[tiab]    143

#30      colonoscop*[tiab]      27346

#31      colonography[tiab]    2034

#32      sigmoidoscopy[tiab]  3941

#33      #21 OR #22 OR #23 OR #24 OR #25 OR #26 OR #27 OR #28 OR #29 OR #30 OR #31 OR #32    254935

#34      #15 AND #20 AND #33         2610

#35      "PSYCHOMETRICS"[Mesh]     69487

#36      "Patient Health Questionnaire"[Mesh]          123

#37      "Behavior Rating Scale"[Mesh]          232

#38      "Patient Reported Outcome Measures"[Mesh]         2460

#39      "Validation Studies"[pt]         92900

#40      questionnaire*[tiab]   452350

#41      psychometr*[tiab]      42299

#42      measure[tiab] 499271

#43      measures[tiab]            647419

#44      instrument*[tiab]        263840

#45      tool[tiab]        422907

#46      tools[tiab]       227210

#47      item[tiab]        82468

#48      items[tiab]      106424

#49      scale[tiab]       626221

#50      scales[tiab]     123247

#51      subscale*[tiab]            38590

#52      validation[tiab]           176761

#53      validity[tiab]   156219

#54      reliability[tiab]            147106

#55      internal consistency[tiab]       25627

#56      convergent[tiab]         26068

#57      discrimina*[tiab]         237704

#58      construct[tiab]            122485

#59      #35 OR #36 OR #37 OR #38 OR #39 OR #40 OR #41 OR #42 OR #43 OR #44 OR #45 OR #46 OR #47 OR #48 OR #49 OR #50 OR #51 OR #52 OR #53 OR #54 OR #55 OR #56 OR #57 OR #58     3217870

#60      #34 AND #59  780 [hits related to instruments]

#61      #34 NOT #59  1830 [hits related to satisfaction as a measurable domain]

**PyscINFO PsycNET 09/01/2019**

**Updated in 19/06/2020**

#1        Index Terms: {Satisfaction} OR {Client Satisfaction} OR {Consumer Satisfaction} OR {Job Satisfaction} OR {Life Satisfaction} OR {Marital Satisfaction} OR {Need Satisfaction} OR {Relationship Satisfaction} OR {Role Satisfaction} OR {Sexual Satisfaction}           55514

#2        Title: satisfaction OR Title: satisfied OR Abstract: satisfaction OR Abstract: satisfied         106081

#3        Title: patient experience OR Title: patients experience OR Title: participant experience OR Title: participants experience          1976

#4        Title: preference*        24566

#5        Title: discomfort OR Abstract: discomfort     8897

#6        Title: burdensome OR Abstract: burdensome           1121

#7        Title: acceptab*          2965

#8        Title: willing*   2720

#9        #1 OR #2 OR #3 OR #4 OR #5 OR #6 OR #7 OR #8 156687

#10      Index Terms: {Health Screening} OR {Health Promotion}     25871

#11      Title: screening OR Abstract: screening         58478

#12      Title: early detection  921

#13      #11 OR #12 OR #13   81649

#14      Title: colorectal           1476

#15      Title: "fecal occult blood test" OR Title: "faecal occult blood test" OR Abstract: "fecal occult blood test" OR Abstract: "faecal occult blood test"         168

#16      Title: "fecal immunochemical test" OR Title: "faecal immunochemical test" OR Abstract: "fecal immunochemical test" OR Abstract: "faecal immunochemical test"           30

#17      Title: stool OR Abstract: stool            501

#18      Title: FOBT OR Title: gFOBT OR Abstract: FOBT OR Abstract: gFOBT           186

#19      Title: colonoscop* OR Title: colonography OR Title: sigmoidoscopy OR Abstract: colonoscop* OR Abstract: colonography OR Abstract: sigmoidoscopy 595

#20      #14 OR #15 OR #16 OR #17 OR #18 OR #19            2273

#21      #9 AND #13 AND #   75

#22      Index Terms: {Questionnaires} OR Index Terms: {Test Construction}            50970

#23      Title: questionnaire* OR Abstract: questionnaire*     255797

#24      Title: psychometr* OR Abstract: psychometr*           51109

#25      Title: measure OR Title: measures OR Abstract: measure OR Abstract: measures   464634

#26      Title: instrument* OR Abstract: instrument*   129473

#27      Title: tool OR Title: tools OR Title: item OR Title: items OR Title: subscale* OR Abstract: tool OR Abstract: tools OR Abstract: item OR Abstract: items OR Abstract: subscale*            311694

#28      Title: validation OR Title: validity OR Abstract: validation OR Abstract: validity     155863

#29      Title: reliability OR Abstract: reliability          79408

#30      Title: "internal consistency" OR Abstract: "internal consistency"      24305

#31      Title: convergent OR Abstract: convergent    17107

#32      Abstract: discrimina* OR Abstract: discrimina*         116759

#33      Title: construct OR Abstract: construct           81208

#34      #22 OR #23 OR #24 OR #25 OR #26 OR #27 OR #28 OR #29 OR #30 OR #31 OR #32 OR #33    1140302

#35      #21 AND #34  26 [hits related to instruments]

#36      #21 NOT #35  49 [hits related to satisfaction as a measurable domain]

**CINAHL EBSCOHost 09/01/2019**

**Updated in 19/06/2020**

S1        (MH "Consumer Satisfaction+")        58,521)

S2        TI satisfaction OR AB satisfaction      60,782

S3        TI satisfied OR AB satisfied    13,547

S4        TI patient experience OR TI patients experience OR TI participant experience OR TI participants experience      4,708

S5        TI preference*             8,443

S6        TI discomfort OR AB discomfort       11,29

S7        TI burdensome OR AB burdensome 1,196

S8        TI acceptab*   3,81

S9        TI willing*        2,108

S10      S1 OR S2 OR S3 OR S4 OR S5 OR S6 OR S7 OR S8 OR S9    131,931

S11      (MH "Health Screening+")     76,563

S12      TI screening OR AB screening            105,034

S13      TI early detection       2,998

S14      S11 OR S12 OR S13    149,374

S15      (MH "Colorectal Neoplasms+")         32,824

S16      (MH "Colonoscopy+")            8,079

S17      (MH "Sigmoidoscopy")          935

S18      TI colorectal    17,747

S19      TI faecal occult blood test OR AB faecal occult blood test OR TI fecal occult blood test OR AB fecal occult blood test       670

S20      TI faecal immunochemical test OR AB faecal immunochemical test OR TI fecal immunochemical test OR AB faecal immunochemical test        322

S21      TI stool OR AB stool   5,004

S22      TI FOBT OR AB FOBT OR TI gFOBT OR AB gFOBT    493

S23      TI colonoscop* OR AB colonoscop* 6,358

S24      TI colonography OR AB colonography         702

S25      TI sigmoidoscopy OR AB sigmoidoscopy     829

S26      S15 OR S16 OR S17 OR S18 OR S19 OR S20 OR S21 OR S22 OR S23 OR S24 OR S25        47,267

S27      S10 AND S14 AND S26          314

S28      (MH "Psychometrics")            21,645

S29      (MH "Questionnaires+")         343,625

S30      (MH "Validation Studies")      101,877

S31      TI questionnaire* OR AB questionnaire*       169,716

S32      TI psychometr* OR AB psychometr* 18,894

S33      TI measure OR AB measure OR TI measures OR AB measures         298,869

S34      TI instrument* OR AB instrument*     73,33

S35      TI tool OR TI tools OR AB tool OR AB tools 129,702

S36      TI item OR TI items OR AB item OR AB items           50,909

S37      TI scale OR TI scales OR AB scale OR AB scales OR TI subscale* OR AB subscale*             179,381

S38      TI validation OR AB validation          38,98

S39      TI validity OR AB validity       54,078

S40      TI reliability OR AB reliability            49,086

S41      TI internal consistency OR AB internal consistency   12,223

S42      TI convergent OR AB convergent      5,57

S43      TI discrimina* OR AB discrimina*      36,969

S44      TI construct OR AB construct             33,755

S45      S28 OR S29 OR S30 OR S31 OR S32 OR S33 OR S34 OR S35 OR S36 OR S37 OR S38 OR S39 OR S40 OR S41 OR S42 OR S43 OR S44          983,978

S46      S27 AND S45 166 [hits related to instruments]

S47      S27 NOT S46   148 [hits related to satisfaction as a measurable domain]

## Annex B. Updated criteria for good measurement properties

| Measurement property | Rating | Criteria |
| --- | --- | --- |
| Structural validity | + | **CTT:**  CFA: CFI or TLI or comparable measure >0.95 OR RMSEA  <0.06 OR SRMR <0.082  **IRT/Rasch:**  No violation of unidimensionality3: CFI or TLI or comparable  measure >0.95 OR RMSEA <0.06 OR SRMR <0.08  *AND*  no violation of local independence: residual correlations  among the items after controlling for the dominant factor <  0.20 OR Q3's < 0.37  *AND*  no violation of monotonicity: adequate looking graphs OR item  scalability >0.30  *AND*  adequate model fit:  IRT: χ2 >0.01  Rasch: infit and outfit mean squares ≥ 0.5 and ≤ 1.5 OR Zstandardized  values > ‐2 and <2 |
|  | ? | CTT: Not all information for ‘+’ reported  IRT/Rasch: Model fit not reported |
|  | - | Criteria for ‘+’ not met |
| Internal consistency | + | At least low evidence4 for sufficient structural validity5 AND  Cronbach's alpha(s) ≥ 0.70 for each unidimensional scale or  subscale6 |
|  | ? | Criteria for “At least low evidence4 for sufficient structural  validity5” not met |
|  | - | At least low evidence4 for sufficient structural validity5 AND  Cronbach’s alpha(s) < 0.70 for each unidimensional scale or  subscale6 |
| Reliability | + | ICC or weighted Kappa ≥ 0.70 |
|  | ? | ICC or weighted Kappa not reported |
|  | - | ICC or weighted Kappa < 0.70 |
| Measurement error | + | SDC or LoA < MIC5 |
|  | ? | MIC not defined |
|  | - | SDC or LoA > MIC5 |
| Hypothesis testing for construct validity | + | The result is in accordance with the hypothesis7 |
|  | ? | No hypothesis defined (by the review team) |
|  | - | The result is not in accordance with the hypothesis7 |
| Cross-cultural validity/ measurement invariance | + | No important differences found between group factors (such  as age, gender, language) in multiple group factor analysis OR  no important DIF for group factors (McFadden's R2 < 0.02) |
|  | ? | No multiple group factor analysis OR DIF analysis performed |
|  | - | Important differences between group factors OR DIF was  found |
| Criterion validity | + | Correlation with gold standard ≥ 0.70 OR AUC ≥ 0.70 |
|  | ? | Not all information for ‘+’ reported |
|  | - | Correlation with gold standard < 0.70 OR AUC < 0.70 |
| Responsiveness | + | The result is in accordance with the hypothesis7 OR AUC ≥ 0.70 |
|  | ? | No hypothesis defined (by the review team) |
|  | - | The result is not in accordance with the hypothesis7 OR AUC <  0.70 |

The criteria are based on e.g. Terwee *et al.* and Prinsen *et al*. (29,30)

AUC = area under the curve, CFA = confirmatory factor analysis, CFI = comparative fit index, CTT

= classical test theory, DIF = differential item functioning, ICC = intraclass correlation coefficient,

IRT = item response theory, LoA = limits of agreement, MIC = minimal important change,

RMSEA: Root Mean Square Error of Approximation, SEM = Standard Error of Measurement, SDC

= smallest detectable change, SRMR: Standardized Root Mean Residuals, TLI = Tucker‐Lewis

index

1 “+” = sufficient, ” –“ = insufficient, “?” = indeterminate

2 To rate the quality of the summary score, the factor structures should be equal across studies

3 unidimensionality refers to a factor analysis per subscale, while structural validity refers to a

factor analysis of a (multidimensional) patient‐reported outcome measure

4 As defined by grading the evidence according to the GRADE approach

5 This evidence may come from different studies

6 The criteria ‘Cronbach alpha < 0.95’ was deleted, as this is relevant in the development phase of

a PROM and not when evaluating an existing PROM.

7 The results of all studies should be taken together and it should then be decided if 75% of the

results are in accordance with the hypotheses

## Annex C. Quality of the instruments development studies

| **Instrument (reference)** | **Design** | | | | | | | **Cognitive interview (CI) study** | | | | **TOTAL PROM DEVELOPMENT** |
| --- | --- | --- | --- | --- | --- | --- | --- | --- | --- | --- | --- | --- |
|  | General design requirements | | | | | Concept elicitation | Total PROM design | General design requirements | Comprehen-sibility | Comprehen-siveness | Total CI study |  |
|  | Clear construct | Clear origin of construct | Clear target population for which the PROM was developed | Clear context of use | PROM developed in sample representing the target population |  |  | CI study performed in sample representing the target population |  |  |  |  |
| CSSQP-Brotons 2019 (32) | V | D | V | D | V | D | D | A | D | D | D | D |
| Patient Satisfaction Survey- Hatoum 2016 (33) | V | D | V | D | D | D | I | NA | NA | NA | NA | I |
| Post procedure questionnaire -Peña 2005 (35) | V | V | V | V | V | D | D | V | D | D | D | D |
| SmGHAA-9m-Sánchez del Río 2005 (34) | V | D | V | D | D | I | I | NA | NA | NA | NA | I |
| Patient satisfaction with screening flexible sigmoidoscopy-Schoen 2000 (36) | V | V | V | D | D | D | D | D | D | NA | D | D |

# V: very good; A: adequate; D: doubtful; I: inadequate; NA: Not applicable

## Annex D. Example of methodological quality assessment and rating of measurement properties

| CSSQP questionnaire(32) | Study quality | Reason for judgement | Measurement property rating | Reason for judgement |
| --- | --- | --- | --- | --- |
| 1.Content validity | D |  | + |  |
| Asking patients-Relevance | D | *Not clear if patients were asked whether each item is relevant or doubtful whether the method was appropriate  *The number of patients in which items were tested is not specified  *Not clear if all group meetings or interviews were recorded and transcribed verbatim  *Not clear what approach was used to analyse data | + | *Based on review ratings as not enough information was provided for the development and validation studies (indeterminate ratings)  *The validation study did not ask patients or professionals about relevance, comprehensiveness and comprehensibility  *At least 85% of items were relevant for the construct, target population, and context of interest.  *It is not clear how response options were consensuated  *Recall period appropriate |
| Asking patients- Comprehensiveness | D | *Method for assessing comprehensiveness was not described  *Number of patients in which items were tested is not specified  *Not clear what approach was used or doubful whether the approach for data analysis was appropriate  *Not clear if two researchers conducted the analysis or it was one | ? | *The validation study did not ask patients or professionals about relevance, comprehensiveness and comprehensibility  * It is not clear wether the authors asked patients about comprehensiveness. |
| Asking patients-Comprehensibility | D | *Doubtful if patients were asked about the comprehensibility of all items and response options  *Number of patients in which items were tested is not specified  *Not clear what approach was used or doubful whether the approach for data analysis was appropriate  *Not clear if two researchers conducted the analysis or it was one | + | *Based on review ratings as not enough information was provided for the development and validation studies (indeterminate ratings)  *Items were appropriately worded, and response options matched the questions |
| Asking experts-Relevance | - | Professionals were not asked |  |  |
| Asking experts- Comprehensiveness | - | Professionals were not asked |  |  |
| 2.Structural validity | A | Exploratory factor analysis conducted but not confirmatory.  Sample size included in the analysis was adequate (n= 505), with more than 100 people and more than 7 times the number of items. | ? | A confirmatory factor analysis was not conducted. They only conducted an exploratory factor analysis. |
| 3.Internal consistency | V | *Internal consistency was calculated for each subscale  *Cronbach’s alpha was calculated for continuous and dichotomic scores | + | Cronbach’s alpha of 0.86 (≥0.7) and Spearman-Brown coefficient of 0.85.  Evidence for good internal consistency comes from one study but with a sample size of 505 |
| 4.Cross-cultural validity | - | Not assessed | - |  |
| 5.Reliability | - | Not assessed | - |  |
| 6.Measurement error | - | Not assessed | - |  |
| 7.Criterion validity | - | Not assessed | - |  |
| 8.Construct validity | - |  | - |  |
| Convergent validity | - | Not assessed | - |  |
| Known groups validity | V | *Subgroups were well described according to sex and educational level.  *The statistical method (multivariate analysis) was appropriate for the hypotheses to be tested | ? | Authors did not define the hypothesis |
| 9.Responsiveness | - | Not assessed | **-** |  |
| Comparison with gold standard | - | Not assessed | - |  |
| Comparison with other instruments | - | Not assessed | - |  |
| Comparison between subgroups | - | Not assessed | - |  |
| Comparison before and after intervention | - | Not assessed | - |  |

V: very good; A: adequate; D: doubtful; I: inadequate; +: sufficient; -: insufficient: ?: indeterminate; +/-: inconsistent.

## Annex F. Information on interpretability of questionnaires

| Questionnaire (reference) | Distribution of scores | Missing items (%) and missing total scores (%) | Floor and ceiling effects | Scores and change scores for (sub)groups | Minimal important change (MIC) or minimal important difference (MID) | Information on response shift |
| --- | --- | --- | --- | --- | --- | --- |
| CSSQP(Brotons 2019) (32) | 79.5% of patients were “satisfied” vs 20.5% were “not satisfied” | Global response rate of 74.8% (378). 8 questionnaires were incomplete and excluded (8/505; 2.1%) | Not identified to eliminate any of the elements. Two items excluded for low item-total correlation (<0.5) | Overall satisfaction was not related to sex, education, marital status or experience of previous colonoscopy. Differences according to sex and studies on Information, Care, Services and facilities. | Not reported | Not reported |
| Patient Satisfaction Survey(Hatoum 2016)(33) | The score was between 0 and 400, where a lower score indicates higher satisfaction  Mean 97.2 SD 75.3 Median 100  Min 0, max 400 | 16 patients (1.32%) did not complete the questionnaire. | Possible floor effect (denoting higher satisfaction) for three of four items related to satisfaction | Score for patients administered sodium picosulfate and magnesium citrat was better than for those administered polyethylene glycol + bisacodyl (50 vs 120, p<0.0001) | Not reported | Not reported |
| Post- procedure questionnaire(Peña 2005)(35) | 10% had an adverse endoscopic experience (score ≥5 on the post-procedure overall satisfaction or unwillingness to repeat the endoscopy) | Not reported | Not reported | Not reported | Not reported | Not reported |
| SmGHAA-9m (Sánchez del Río 2005)(34) | Not reported | 30 patients (30/485; 6.2%) did not complete the questionnaire | Not reported | Not reported | Not reported | Not reported |
| Screening Flexible Sigmoidoscopy Assessment Questionnaire (Schoen 2000)(36) | Mean score (SD) for overall satisfaction: 1.76 Mean score (SD) for pain and discomfort: 1.76 (0.45).  Scores from 1 to 5, being the lower scores more favorable. | The percentage of missing responses for each item ranged from 1.4% to 2.6%. | Not reported | Overall satisfaction men vs women: 1.7 vs 1.8, p<0.001 Pain and discomfort men vs women: 2.0 vs 2.4 (p<0.001). | Not reported | Not reported |

## Annex E. Information on feasibility of questionnaires

| **Feasibility aspects** | **CSSQP** (Brotons 2019)(32) | **Patient Satisfaction Survey** (Hatoum 2016)(33) | **Post- procedure questionnaire** (Peña 2005)(35) | **SmGHAA-9m** (Sánchez del Río 2005)(349 | **Screening Flexible Sigmoidoscopy Assessment Q** (Schoen 2000)(36) |
| --- | --- | --- | --- | --- | --- |
| Patient’s comprehensibility | Not reported | Not reported | Not reported | Not reported | Not reported |
| Clinician’s comprehensibility | Not reported | Not reported | Not reported | Not reported | Not reported |
| Type and ease of administration | Self reported and telephone reminder | Self reported | Self reported | By telephone | Self reported |
| Length of the instrument | 3 pages | Not reported | 1 page | Not reported | 5 pages |
| Completion time | Not reported | Not reported | Not reported | Not reported | Not reported |
| Patient’s required mental and physical ability level | Not reported | Not reported | Patients with cognitive impairment were excluded | Not reported | Not reported |
| Ease of standardization | Not reported | The raw score of each item was transformed to a range from 0 to 100 to standardize items. | Not reported | Not reported | Negative items were recorded so that lower numbers indicated a more favorable response. |
| Ease of score calculation | Satisfaction scale was based on a rating scale ranging from 1 (poor) to 5 (excellent). Perceived safety scale was composed of three items with dichotomous response options: yes (1), no (2). The CSSQP score was calculated if 50% of questions were responded. | Scores for each item were summed to generate a total score, where lower scores indicate higher satisfaction. | Not reported | Scores for items 1 to 7 (from 1 to 5) were summed to generate a total score (from 7 to 35). The higher the score, the greater the level of satisfaction. | Satisfaction scale averages responses to 17 of the 18 items (pretest anxiety item was found not related to overall satisfaction) and weight responses across all seven domains. Pain and discomfort scale averages responses to the three items pertaining to the domain pain and discomfort. |
| Copyright | Not reported | Not reported | Not reported | Not reported | Not reported |
| Cost of an instrument | Not reported | Not reported | Not reported | Not reported | Not reported |
| Required equipment | Not reported | Not reported | Not reported | Not reported | Not reported |
| Availability in different settings | Available in English and Spanish | Only available in English. Authors were contacted unsuccessfully to ask for any translation. | Only available in English. Authors were contacted unsuccessfully to ask for any translation. | Available in English and Spanish | Only available in English. Authors were contacted unsuccessfully to ask for any translation. |
| Regulatory agency’s requirement for approval | Not reported | Not reported | Not reported | Not reported | Not reported |
